# Mesenchymal stem cells induce dendritic cell immune tolerance via paracrine hepatocyte growth factor to alleviate acute lung injury

**DOI:** 10.1186/s13287-019-1488-2

**Published:** 2019-12-04

**Authors:** Zhonghua Lu, Wei Chang, Shanshan Meng, Xiuping Xu, Jianfeng Xie, Fengmei Guo, Yi Yang, Haibo Qiu, Ling Liu

**Affiliations:** Department of Critical Care Medicine, Zhongda Hospital, School of Medicine, Southeast University, 87 Dingjia Bridge, Hunan Road, Gu Lou District, Nanjing, 210009 China

**Keywords:** Acute respiratory distress syndrome (ARDS), Acute lung injury (ALI), Dendritic cells, Hepatocyte growth factor (HGF), Mesenchymal stem cell (MSC), Akt

## Abstract

**Background:**

Mesenchymal stem cells (MSCs) have been shown to alleviate acute lung injury (ALI) via paracrine hepatocyte growth factor (HGF) and to induce the differentiation of dendritic cells (DCs) into tolerogenic dendritic cells (DCregs) and participate in the immune response. However, whether MSCs induce the production of DCregs by secreting HGF to alleviate early ALI remains unclear. We observed that the protective effect of mouse bone marrow-derived MSCs against lipopolysaccharide (LPS)-induced ALI was achieved by inducing mature DCs (mDCs) to differentiate into DCregs, and its mechanism is related to the activation of the HGF/Akt pathway.

**Methods:**

MSCs or MSCs with overexpression or knockdown of HGF were cocultured with DCs derived from mouse bone marrow using a Transwell system for 3 days. Moreover, we used MSCs or MSCs with overexpression or knockdown of HGF to treat LPS-induced ALI mice for 24 h. Flow cytometry was performed to measure the phagocytosis, accumulation, and maturation of DCs, as well as proliferation of T cells. Lung injury was estimated by lung wet weight to body weight ratio (LWW/BW) and histopathological analysis. Furthermore, we used the Akt inhibitor MK-2206 in a coculture system to elucidate the role of the HGF/Akt pathway in regulating the differentiation of DCs into regulatory DCs and relieving lung injury in early ALI mice.

**Results:**

Immature DCs (imDCs) were induced to mature after 24 h of LPS (50 ng/ml) stimulation. MSCs or HGF induced the differentiation of mDCs into regulatory DCs characterized by low expression of MHCII, CD86, and CD40 molecules, strong phagocytic function, and the ability to inhibit T cell proliferation. The effect of MSCs on DCregs was enhanced with the increase in HGF secretion and was weakened with the decrease in HGF secretion. DCregs induced by recombinant HGF were attenuated by the Akt inhibitor MK-2206. Lung DC aggregation and mDC ratio increased in LPS-induced ALI mice, while treatment with MSCs decreased lung DC aggregation and maturation and alleviated lung pathological injury. High expression of the HGF gene enhanced the above effect of MSCs, while decreased expression of HGF weakened the above effect of MSCs.

**Conclusions:**

MSCs alleviate early ALI via paracrine HGF by inducing mDCs to differentiate into regulatory DCs. Furthermore, the mechanism of HGF-induced differentiation of mDCs into DCregs is related to the activation of the Akt pathway.

## Background

At present, acute respiratory distress syndrome (ARDS) remains a relatively common and lethal or disabling syndrome despite decades of improvements in supportive and pharmacological interventions [1–4]. However, there may be heterogeneity of disease mechanisms in ARDS due to different causes, possibly contributing to the failure of countless interventions to improve outcomes in clinical trials [5]. Compared with extrapulmonary ARDS, pathological inflammatory infiltration and inflammatory markers of pulmonary ARDS are more obvious and more sensitive to treatment response [6]. An uncontrolled inflammatory response is the key mechanism of pulmonary ARDS pathogenesis [7]. Innate immune cell-mediated damage to the alveolar endothelial and epithelial barrier, resulting in increased accumulation of protein-rich edema fluid in the interstitium and alveoli, is the initial response of the lungs to injury in the exudation phase of ARDS [8–10]. Dendritic cells (DCs) are the most important antigen-presenting cells and play a key role in the initiation of primary immune responses and the induction of tolerance in the pulmonary immune response network [11–13].

Previous studies have shown that the number and maturity of conventional DCs (cDCs) in the lungs of ALI mice are significantly increased, and promoting the maturation of pulmonary DCs can aggravate pulmonary inflammatory reactions and pathological injury [14–16]. Therefore, mature DCs (mDCs) play an important role in the pathophysiology of early ALI, and regulating the immune function of mDCs may have clinical significance in the treatment of ARDS.

Under certain conditions (such as drugs, soluble cytokines, stem cells, and changes in the local microenvironment), mDCs can be induced into regulatory DCs (DCregs), whose main role is to maintain immune tolerance and negatively regulate the effects of the immune response. Recently, studies have shown that mesenchymal stem cells (MSCs) have a superior immunomodulatory capacity to induce the differentiation of DCs into DCregs [17–21], which have a stable phenotype even under lipopolysaccharide (LPS) stimulation [19]. A growing number of studies have also provided convincing data on the beneficial effects of MSCs in treating LPS-induced acute lung injury (ALI) [22–25]. However, it is not clear whether MSCs improve the early lung injury of ALI by inducing mDCs to differentiate into DCregs.

Our previous studies provided reliable data on the protective effects of hepatocyte growth factor (HGF) secreted by MSCs on ALI in vitro and in vivo [26, 27]. Several studies have shown that HGF-treated DCs were characterized by increased expression of programmed death ligand 1 (PD-L1) and the ability to promote the development of IL-10-secreting regulatory T cells [28]. HGF also inhibits DC migration by binding to the HGF receptor-mesenchymal transition factor (c-Met) on the surface of DCs [29], which promotes downstream activation of the phosphatidylinositol 3-kinase/Akt pathway, inhibits antigen presentation, and downregulates surface markers for T cell activation [30, 31]. Whether the Akt pathway is an important link in HGF-induced differentiation of mDCs into DCregs remains unknown.

The aim of this study was to determine whether the protective effect of MSCs against LPS-induced ALI was achieved by inducing mDCs to differentiate into DCregs and whether the mechanism is related to the activation of the HGF/Akt pathway. We used coculture experiments to test the phenotype, phagocytosis, stimulation of T cell proliferation, and cytokine secretion of DCs. To investigate the effects and potential mechanisms of MSCs inducing mDCs to convert into DCregs, we used MSCs with low expression (shHGF-MSCs) and overexpression (HGF-MSCs) of HGF genes cocultured with mDCs and then explored the potential mechanisms of the protective effects of HGF on inducing mDCs to convert into DCregs by the Akt inhibitor MK2206. To study the effect of HGF in MSCs in the ALI mouse model, we used HGF-MSCs and shHGF-MSCs to treat LPS-induced ALI mice and evaluate lung pathological injury, DC phenotype, pulmonary edema, etc. This study provides an immunological explanation for the reduction of LPS-induced lung injury by MSCs via paracrine HGF.

## Methods

### Mice

Specific-pathogen-free male BALB/c and C57BL/6 mice (age 6–8 weeks, weight 20–25 g) were purchased from the Laboratory Animal Center of Yangzhou University (Yangzhou, China). All animal experiments were carried out in accordance with the National Institutes of Health Guide for the Care and Use of Laboratory Animals. All of the experimental procedures were approved by the Southeast University Ethics Committee.

### MSC culture

Mouse MSCs and DCs were used in the present study. MSCs were purchased from Cyagen Biosciences, Inc. (Guangzhou, China). The supplier identified MSCs based on the cell surface phenotype and pluripotency. Fluorescein-conjugated monoclonal antibodies, including anti-CD29, anti-CD44, anti-CD117, anti-Sca-1, anti-CD31, and anti-CD45, were used. The multipotent potential of MSCs for differentiation along the adipogenic, osteogenic, and chondrogenic lineages was determined by staining with Oil Red O, Alizarin red, and toluidine blue, respectively, followed by culture in adipogenic, osteogenic, and chondrogenic differentiation media, respectively (Cyagen Biosciences, Inc.) for 2 to 3 weeks, thus verifying their identity as mouse MSCs. MSCs were cultured in DMEM/F12 (Wisent, Nanjing, China) containing 10% fetal bovine serum (FBS) (Wisent, Nanjing, China) and grown in a humidified 5% CO2 incubator at 37 °C.

### Generation of mouse BM-derived DCs

Bone marrow (BM)-derived DCs were generated as previously described with minor modifications [32]. BM cells were extracted from the medullary cavity of the femur and tibia. The erythrocytes were lysed using Lysing Buffer (BD Pharm Lyse™, USA), washed three times in PBS, and cultured in 100-mm dishes with 2 × 10^6^ cells containing RPMI-1640 (Wisent, Nanjing, China) medium supplemented with 10% FBS (Wisent, Nanjing, China), 40 ng/ml recombinant murine granulocyte–macrophage colony-stimulating factor (GM-CSF; NOVUS), and 40 ng/ml recombinant murine interleukin-4 (IL-4; NOVUS) in a humidified 5% CO2 incubator at 37 °C. For the isolation of imDCs, non-adherent cells were gently washed out on day 3, and the remaining loosely adherent cell clusters were collected and purified by anti-CD11c micromagnetic beads (Miltenyi Biotec) on day 6. Purified imDCs cultured for an additional 24 h under the stimulation of 50 ng/ml bacterial lipopolysaccharide (LPS; Sigma-Aldrich) were used as mDCs. The purity of the cells was greater than 90%. Cytofluorimetric analysis was performed to evaluate the DC maturation phenotype (CD40, CD86, and MHCII).

### Reagent treatments

Before some experiments, purified imDCs cultured for an additional 24 h under the stimulation of 50 ng/ml bacterial LPS (Sigma-Aldrich) were used as mDCs. To determine the roles and mechanisms of HGF, we introduced recombinant murine HGF (50 ng/ml, R&D Systems, USA) into mature dendritic cells (mDCs) containing 5% FCS and 20 ng/ml GM-CSF RPMI-1640 medium. In addition, phosphate-buffered saline (PBS) was used as a negative control, and the Akt inhibitor MK-2206 (5 μmol/l, Selleck) was used to inhibit the activation of the Akt pathway 3 h before HGF treatment of mDC. The drug concentrations used were according to our preliminary experiments.

### Production of lentiviral vectors and transduction of MSCs

MSCs and 293FT cells were purchased from Cyagen Biosciences, Inc. (Guangzhou, China) as previously described. MSCs from passages 4–7 were used for transduction and HGF gene overexpression and knockdown experiments. HGF gene overexpression and reduced expression were achieved using lentiviral vectors, and lentiviruses for overexpression and reduced expression specific for enhanced green fluorescent protein (EGFP) (PDS087 and PDS019) were used as negative controls. The lentiviruses were packaged in 293T cells (Cyagen Biosciences, Inc.) with the aid of three packaging plasmids, and then a higher or lower lentivirus titer was obtained. The MSCs were transfected and screened by the antibiotic blasticidin for 7 to 14 days. Subsequently, MSCs carrying empty vectors and EGFP (NC-HGF-MSCs, NC-shHGF-MSCs) or MSCs carrying both the HGF gene and EGFP (HGF-MSCs, shHGF-MSCs) were harvested.

### DC and MSC coculture

MSCs were seeded onto the lower chamber of a six-well Transwell system (0.4-mm pore size membrane; Corning, Cambridge, MA, USA). When MSCs, NC-HGF-MSCs, HGF-MSCs, NC-shHGF-MSCs, and shHGF-MSCs were attached, the medium was changed with RPMI-1640 medium containing 5% FCS (Wisent, Inc., Nanjing, China) and 20 ng/ml GM-CSF, 5 × 105 mDCs were seeded onto the upper chamber per well in six-well plates. The ratio of MSCs/mDCs is 10:1. After cocultivation for 72 h, the upper chamber cells were taken for examination or applied to the next experiment.

### ALI model

The ALI model was induced as previously described with minor modifications [33]. Briefly, mice were intraperitoneally injected with 50 mg/kg pentobarbital. LPS (5 mg/kg) (Sigma-Aldrich) was delivered to the lungs through a tracheostomy, and the incision was sewed up. Mice were returned to the cage until fully awake.

### Experimental groups and sample acquisition

The mice were randomly assigned to one of the following groups (*n* = 5 mice per group): control group (Con), mice were given the same amount of 0.9% normal saline (NS) or PBS at the corresponding time; ALI group (ALI), mice received 5 mg/kg LPS to establish the ALI model; MSC + ALI group (MSCs), mice received MSCs (500,000 cells in 150 μl PBS) via the tail vein 6 h after LPS; NC-HGF-MSC + ALI group (NC-HGF-MSCs), mice received NC-HGF-MSCs (500,000 cells in 150 μl PBS) via the tail vein 6 h after LPS; HGF-MSC + ALI group (HGF-MSCs), mice received HGF-MSCs (500,000 cells in 150 μl PBS) via the tail vein 6 h after LPS; NC-shHGF-MSC + ALI group (NC-shHGF-MSCs), mice received NC-shHGF-MSCs (500,000 cells in 150 μl PBS) via the tail vein 6 h after LPS; shHGF-MSC + ALI group (shHGF-MSCs), mice received shHGF-MSCs (500,000 cells in 150 μl PBS) via the tail vein 6 h after LPS. The mice were sacrificed after 24 h; lung tissue was collected for single cell isolation and histological examination in accordance with slightly modified previous methods [14].

### Evaluation of lung edema

Lung wet weight to body weight ratios (LWW/BW), which reflect the severity of lung vascular permeability and lung edema, were obtained for the control, ALI, MSC, NC-HGF-MSC, HGF-MSC, NC-shHGF-MSC, and shHGF-MSC groups.

### Lung histopathology

The right upper lobe was embedded in paraffin and sagittally sliced at 5-μm thickness. The sections were stained with hematoxylin and eosin. Edema, alveolar and interstitial inflammation and hemorrhage, atelectasis, necrosis, and hyaline membrane formation were each scored using a 0 to 4-point scale. The severity of lung injury was calculated as the sum of the scores as previously described [26].

### Flow cytometry

Lung cell isolation and the measurement of the accumulation and maturation of DCs by flow cytometry were performed as previously described [16]. For phenotypic analysis of cell surface marker expression, cells were harvested, resuspended in PBS, incubated for 15 min with FcR blocking reagent, and then incubated for 15 min with PE-, APC-, PE-Cy7-, PerCP-, or FITC-conjugated monoclonal antibodies (mAbs) on ice. DCs were stained with antibodies against CD11c, CD40, CD86, CD11b, MHCII (Miltenyi Biotec, Bergisch Gladbach, Germany), PD-L1 (BD Pharmingen), and IL-27 (IL-27p28; BioLegend, USA). Mouse IgG1 isotype control antibodies were used in parallel as negative controls. Carboxyfluorescein diacetate succinimidyl ester (CFSE) (BD Pharmingen) was used in T cells, and FITC-dextran was used to examine the phagocytosis of DCs. The stained cells were washed twice and resuspended in cold buffer and then analyzed by flow cytometry (FACSCalibur; NovoCyte), and the results were processed using NovoExpress software. The results are expressed as the percentage of positively stained cells relative to the total cell number.

### Cytokine analysis

DCs were separated and washed after coculture with MSCs for 72 h and then cultured for 24 h. The supernatant was collected, and the concentration of interleukin-12 (IL-12), transforming growth factor beta (TGF-β), and interleukin-10 (IL-10) secreted by DCs was determined by enzyme-linked immunosorbent assay (ELISA, RayBiotech). Quantitative analysis of HGF was performed on supernatants derived from MSC cultures by ELISA according to the manufacturer’s instructions (ELISA, RayBiotech). HGF concentrations were determined using a standard curve constructed by titration of the HGF standard.

### Endocytosis assay

To determine the phagocytic capacity of DC, imDC, mDC, HGF-DC, MSC-DC, NC-HGF-MSC-DC, NC-shHGF-MSC-DC, HGF-MSC-DC, or shHGF-MSC-DC was incubated at 37 °C or at 4 °C as a negative control for 4 h with FITC-conjugated OVA (FITC-OVA; AnaSpec) at a final concentration of 100 ng/ml containing RPMI-1640 with 10% FCS, washed twice with ice-cold PBS containing 0.5% bovine serum albumin (BSA; BioFroxx), and then resuspended in cooled PBS for immediate flow cytometry.

### Mitogen proliferative assay

CFSE-labeled solenocytes (5 × 10^5^ cells/well) were incubated with 5 μg/ml concanavalin A (ConA; Sigma-Aldrich) or cocultured with allogenic DCs (mDCs, MSC-DCs, NC-HGF-MSC-DCs, NC-shHGF-MSC-DCs, HGF-MSC-DCs, or shHGF-MSC-DCs, 5 × 10^4^ cells/well) in a total volume of 0.2 ml medium in 96-well U-bottom plates.

### Western blot analysis

For the HGF/c-Met/Akt assay, phosphorylated Akt, total Akt, and phosphorylated or total c-Met were measured by Western blot analysis. Proteins were separated by sodium dodecyl sulfate–polyacrylamide gel electrophoresis and transferred to polyvinylidene difluoride (PVDF) membranes. The membranes were incubated with primary antibodies against c-Met, HGF (1:1000; Abcam), phospho-c-Met, Akt, and phospho-Akt (1:1000; Cell Signaling) at a 1:1000 dilution at 4 °C overnight. The membranes were incubated with secondary antibody for 1 h at room temperature. Immunoblots were visualized using enhanced chemiluminescence (ECL; Thermo Scientific). The expression levels from whole cell extract were normalized against that of β-actin.

### Statistical analysis

All statistical analyses were performed using SPSS 23.0 software and GraphPad Prism 7.0. One-way analysis of variance (ANOVA) or two-tailed Student’s *t* test was used to determine the significance between the groups. Data are expressed as the mean ± standard deviation (SD). *P* < 0.05 was considered significant.

## Results

### LPS induces the differentiation of imDCs into mDCs

**Fig. 1:**
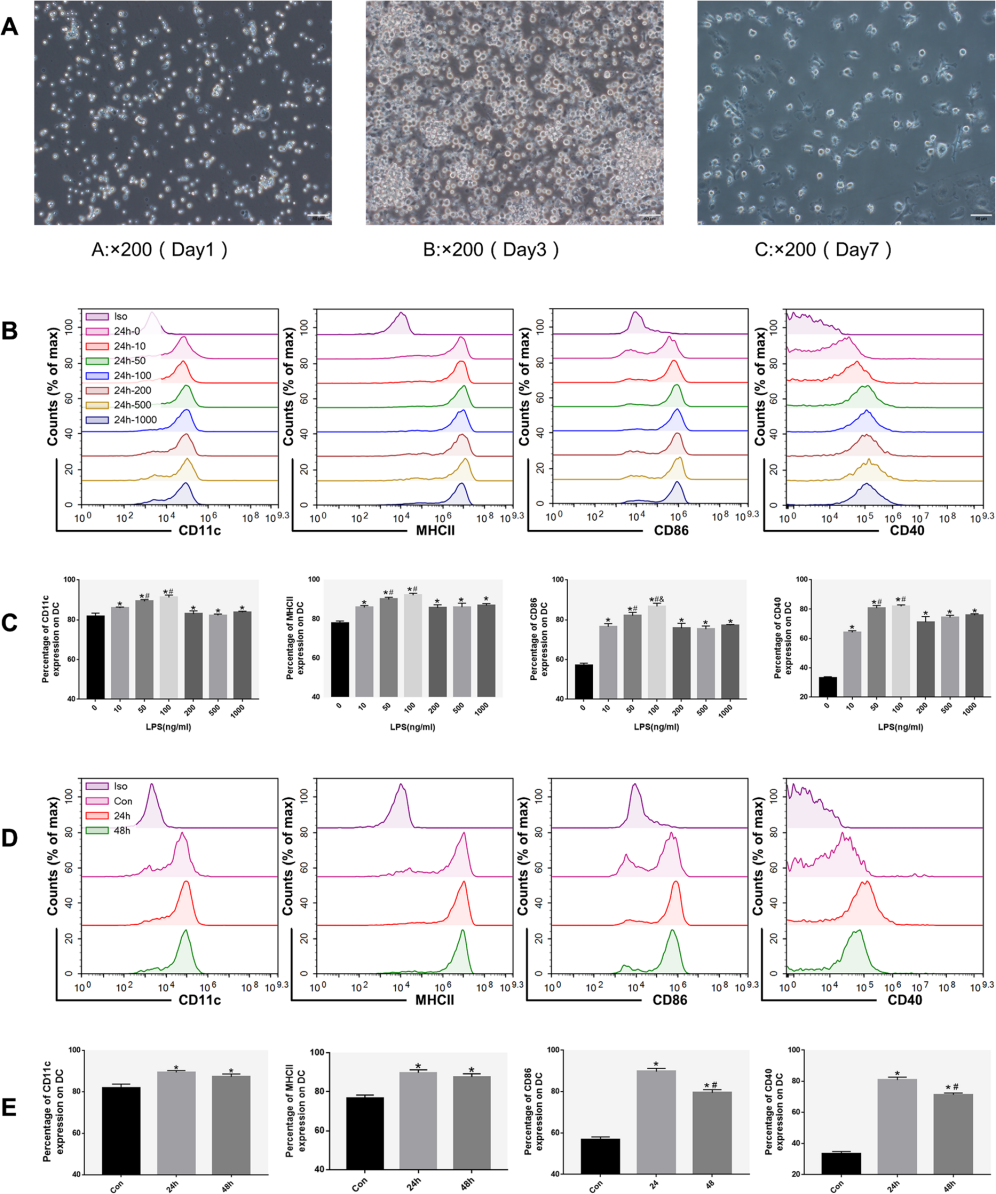
Induction and identification of DCs. **a** The morphology of DCs. Cell morphology on days 1, 3 (left and middle, monocytes in the presence of GM-CSF and IL-4), and 7 (right, imDC cultured for 24 h under LPS stimulation) (×200 magnification). **b** Immunophenotype analysis of DCs (expression of MHCII, CD86, and CD40 in DCs cultured for 24 h in the presence of LPS at concentrations ranging from 0 to 1000 ng/ml). **c** The percentage of DCs positive for MHCII, CD86, and CD40 after incubation for 24 h with LPS at concentrations ranging from 0 to 1000 ng/ml. **d** Immunophenotype analysis of DCs (expression of MHCII, CD86, and CD40 on DCs after culture for 0 h, 24 h, and 48 h with an LPS concentration of 50 ng/ml). **e** The percentage of DCs expressing MHCII, CD86, and CD40 after 0 h, 24 h, and 48 h cultured at an LPS concentration of 50 ng/ml. *n* = 3. **c** *P < 0.05 versus LPS 0 ng/ml group; #*P* < 0.05 versus LPS 10 ng/ml group; &*P* < 0.05 versus LPS 50 ng/ml group. **e** *P < 0.05 versus Con group; #*P* < 0.05 versus 24 h group. Data are expressed as mean ± SD. Each experiment was repeated three times

Mouse BM-derived DCs showed typical characteristics of DCs on day 3, becoming clustered adherent cells and showing various protruding veils, and the typical DC traits became more apparent on the 7th day (Fig. 1a). CD11c^+^ DCs reached over 90% purity after magnetic bead sorting. imDCs were treated with LPS (0–1000 ng/ml) for 0, 24, and 48 h. The LPS-induced mDC phenotype marked by the expression of MHCII, CD86, and CD40 was positively dose-dependent when LPS concentrations were below 50 ng/ml (Fig. 1b, c), but the percentage of cells expressing the mature phenotype was highest at 24 h (Fig. 1d, e). imDCs were induced to mature after 24 h of 50 ng/ml LPS stimulation.

### MSCs and rhHGF induce mDCs to convert into DCregs

**Fig. 2:**
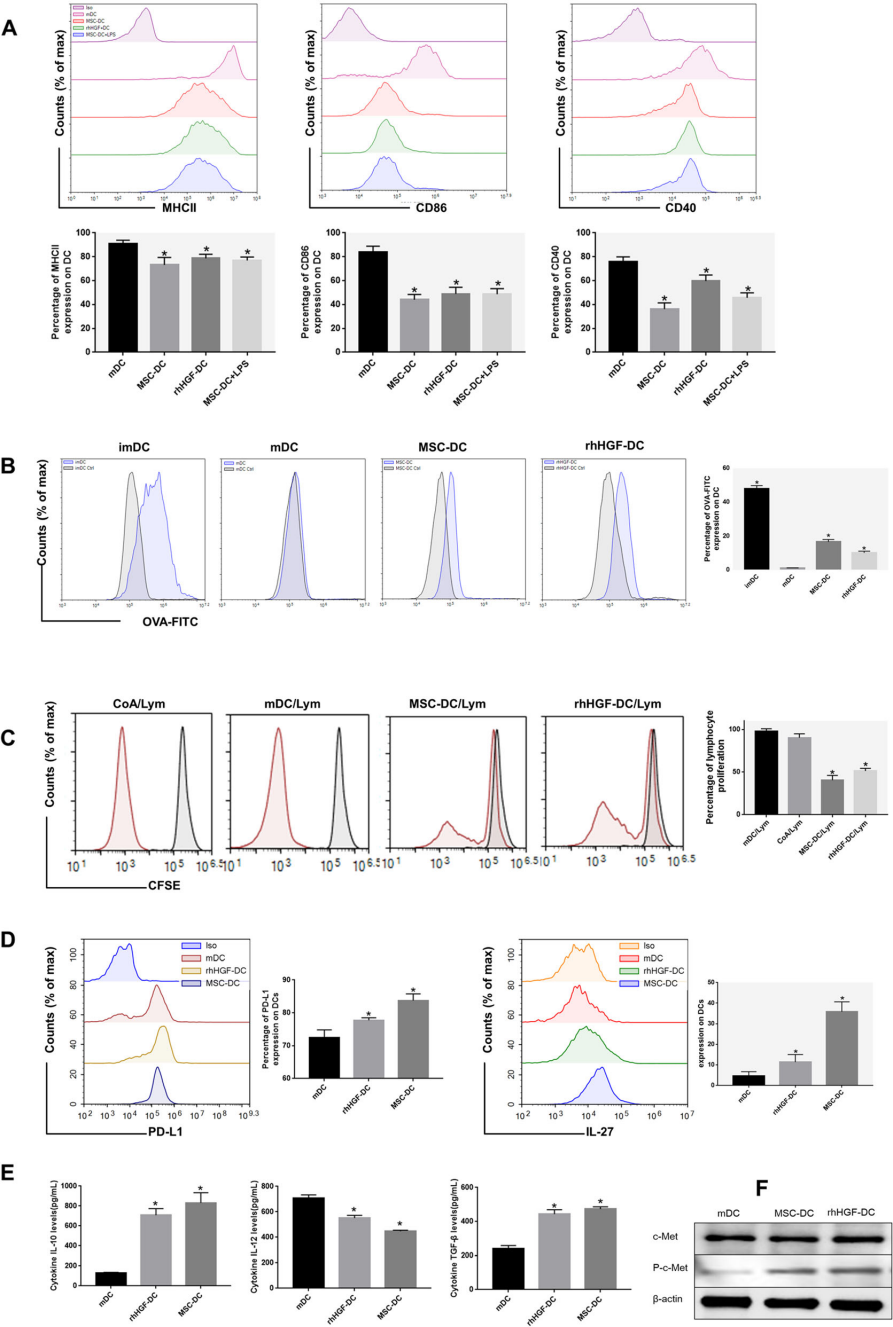
Effects of MSCs and rhHGF on mDC differentiation. **a** Immunophenotype analysis of DCs (expression of MHCII, CD86, and CD40 in mDCs cultured for 72 h in the presence of MSC or rhHGF, and expression of MHCII, CD86, and CD40 in MSC-induced DCs after LPS stimulation for 24 h). Percentage of MHCII, CD86, and CD40 expression on DCs after 72 h of incubation with or without MSC or rhHGF. **b** Phagocytic ability analysis of DCs (expression of OVA-FITC in DCs and percentage of OVA-FITC-positive cells of DCs). **c** Lymphocyte proliferation stimulated by mitotic proenzyme ConA, mDCs, MSC-DCs, or rhHGF-DCs. Splenocytes from normal BALB/c mice were used as responder cells in the mitogen proliferative assay. mDCs, MSC-DCs, rhHGF-DCs, or ConA were used as stimulators. Splenocytes from normal mice served as controls. The proliferative responses were assessed by CFSE labeling and FACS (gray line, unstimulated spleen cell). **d** Immunophenotype analysis of DCs (expression of PD-L1 and IL-27 in mDCs cultured for 72 h in the presence of MSC or rhHGF). Percentage of PD-L1 and IL-27 expression in DCs after 72 h of incubation with or without MSC or rhHGF. **e** Cytokine secretion profiles of mDCs, MSC-DCs, and rhHGF-DCs after culture for 24 h. **f** Changes in c-met and phosphorylated c-met levels in dendritic cells cultured for 72 h in the presence of MSCs or rhHGF (*n* = 3, *P < 0.05 versus mDC group; data are expressed as mean ± SD). Each experiment was repeated times

Interestingly, in contrast to the expression levels in mDCs, phenotype analysis (Fig. 2a) showed that MSC- or rhHGF-treated mDCs expressed less functional markers, such as MHCII, CD86, and CD40, and were similar to imDCs. However, in contrast to imDCs, the addition of LPS to these cells could not restore the expression of the above functional markers, indicating the MSCs-induced mDCs to differentiate into a novel DC population (regulatory DCs) with a more stable phenotype than imDCs. Additionally, compared to mDCs, these novel DCs exhibited stronger phagocytic capacity similar to imDCs (Fig. 2b). We also investigated whether MSC-DCs had an immunomodulatory capacity. When CFSE-labeled splenocytes, used as responders, were cocultured with mDCs, MSC-DCs, and rhHGF-DCs, MSC-DCs and rhHGF-DCs had the weakest effect on stimulating lymphocyte activation (Fig. 2c). Furthermore, MSC- or HGF-induced DCs expressed more immunosuppressive molecules PD-L1 and IL-27 than mDCs (Fig. 2d). Moreover, after culturing DCs independently for another 24 h after thorough washes, we observed that IL-10 and TGF-β were increased in the MSC-DC and rhHGF-DC groups, and IL-12 was decreased (Fig. 2e). In the process of MSC- or rhHGF-induced mDC immune tolerance, the phosphorylation of the HGF-specific receptor c-Met protein was significantly increased (Fig. 2f). The results showed that the immunomodulatory properties of MSC-regulated DCs may depend on paracrine HGF in vitro.

### HGF expression in genetically modified MSCs

**Fig. 3:**
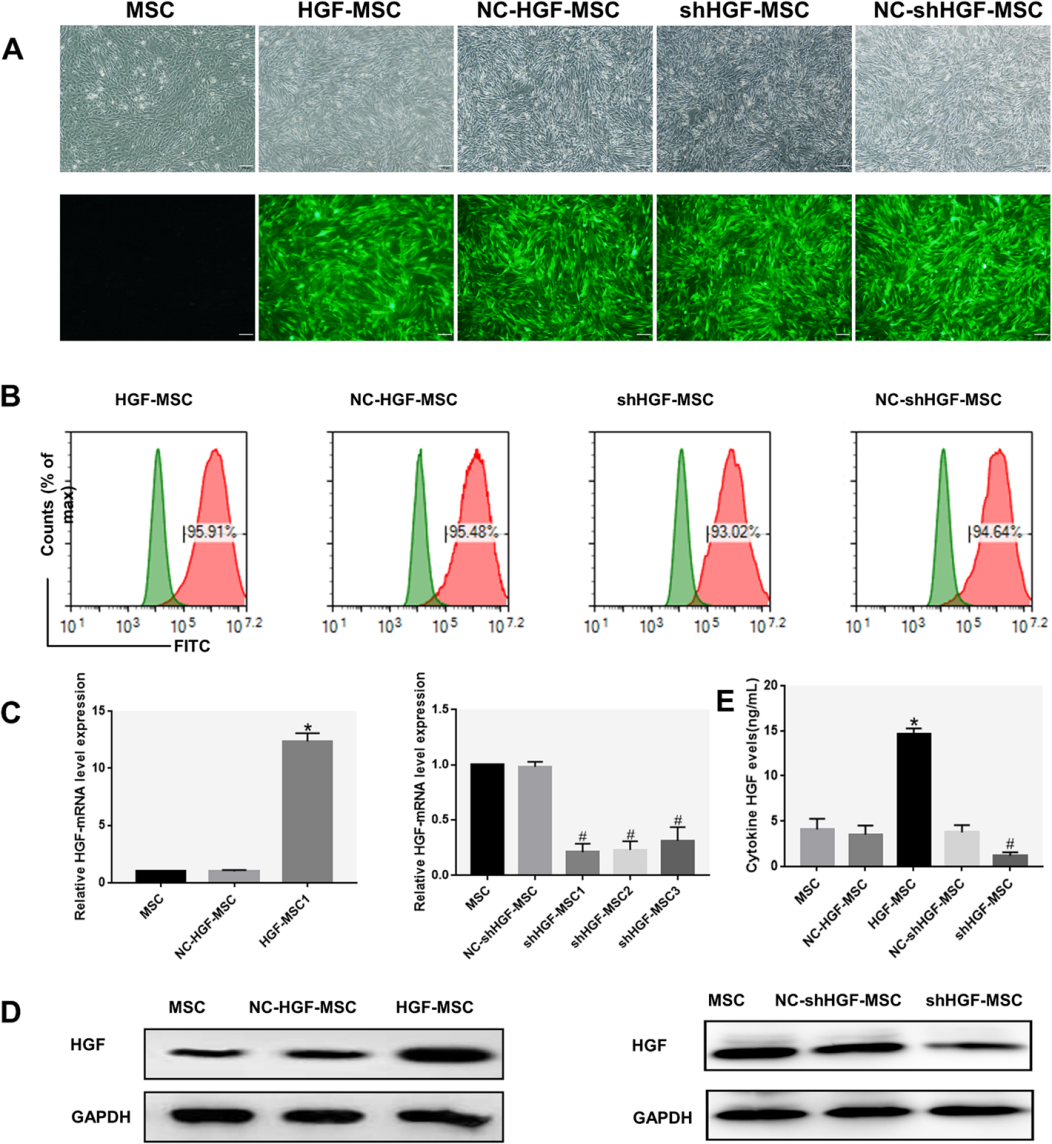
Long-term transgene expression efficiency in MSCs after lentiviral vector transduction. **a** The MSCs transduced separately with PDS087-EGFP (NC-MSCs), CL1017-PDS087_pL6-CMV-EGFP-IRES-HGF (HGF-MSCs), PDS019-EGFP (NC-shHGF-MSCs), and VL1012_pL-m-HGF-shRNA-1173 (shHGF-MSCs) lentiviral vectors cultured for 20 passages and observed with light microscopy (top) and fluorescence microscopy with green fluorescent protein (bottom), ×200. **b** The percentage of GFP-positive cells analyzed by flow cytometry at passage 20 after transduction (Based on untransfected MSCs in the green line, gates on the panel in the red line represent transfection-positive cells). **c** Quantitative real-time PCR analysis of HGF mRNA expression in MSCs after transduction. **d** The expression of HGF protein in MSCs after transduction evaluated using Western blot analysis. **e** The cytokine HGF secreted by MSCs after transduction cultured for 48 h detected by ELISA (*n* = 3, *P < 0.05 versus NC-HGF-MSC; #*P* < 0.05 versus NC-shHGF-MSC; data are expressed as mean ± SD). Each experiment was repeated three times

Our previous research shows that changes in HGF secreted by MSCs have been observed [34]. Whether MSCs induce mDCs to convert into DCregs by secreting HGF remains unclear. To further determine whether the inhibitory effect of MSC-DCs was mediated by secreting HGF, HGF was overexpressed or knocked down by transducing MSCs with HGF or RNAi retroviral vectors. Fluorescence microscopy (Fig. 3a) and flow cytometry (Fig. 3b) images showed the expression of EGFP. The transduction efficiency was over 90% and was well maintained over 20 passages. The expression of HGF mRNA in the HGF-MSC group was approximately 12-fold greater than that of the MSC group, which was fivefold higher than that in the knockdown group (Fig. 3c). HGF protein expression was also increased in the HGF-MSC group while it was decreased in the shHGF-MSC group (Fig. 3d) and resulted in an approximately threefold increase in the HGF secreted into the culture media while the opposite result was observed in the shHGF-MSC group (Fig. 3e). Therefore, all of the data suggested that the HGF gene of MSCs was overexpressed or knocked down at the mRNA and protein levels.

### Differentiation of mDCs into DCregs is attributed to MSCs secreting HGF

**Fig. 4:**
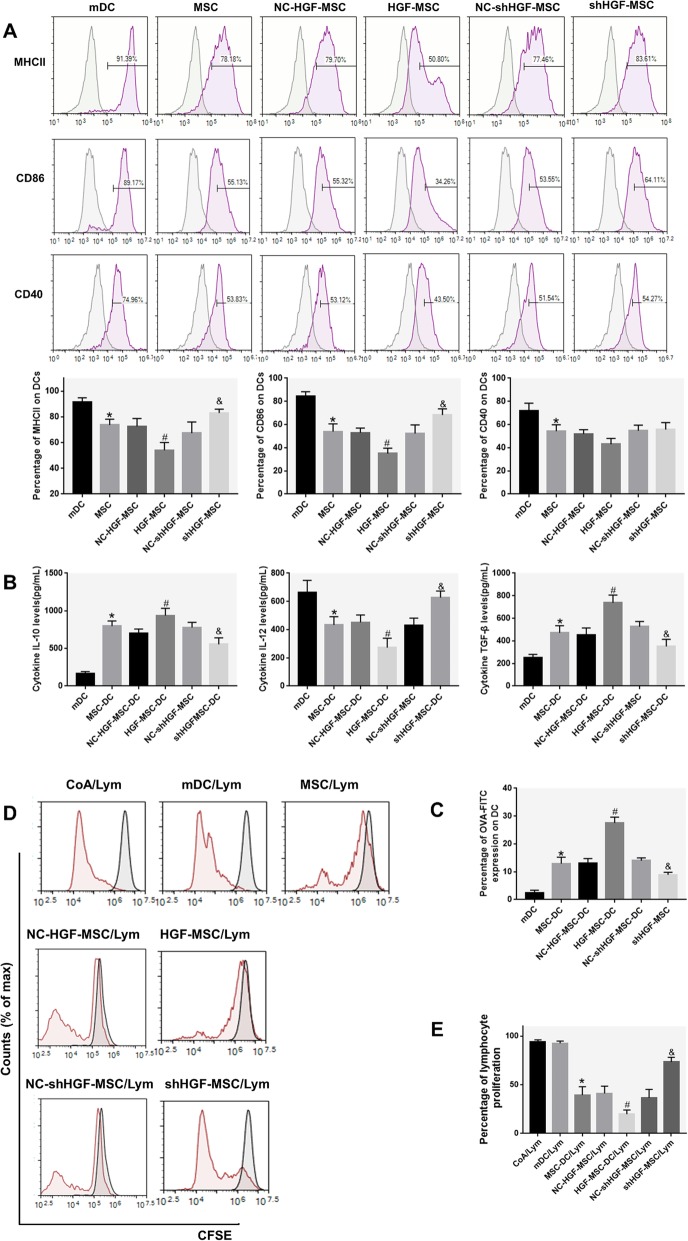
HGF plays a role in the MSC-induced differentiation of DCs. **a** Immunophenotype analysis of DCs (expression of MHCII, CD86, and CD40 on mDCs cultured for 72 h in the presence of MSCs or MSCs after transduction). The percentage of DCs positive for MHCII, CD86, and CD40 after coculture for 72 h with MSCs or transduced MSCs. **b** Cytokine secretion profiles of mDCs, MSC-DCs, NC-HGF-MSC-DCs, HGF-MSC-DCs, NC-shHGF-MSC-DCs, and shHGF-MSC-DCs after culture for 24 h. **c** Phagocytic ability analysis of MSC-induced DCs (percentage of OVA-FITC-positive cells). **d**, **e** Lymphocyte proliferation stimulated by ConA, mDCs, MSC-DCs, NC-HGF-MSC-DCs, HGF-MSC-DCs, NC-shHGF-MSC-DCs, and shHGF-MSC-DCs. Normal BALB/c mouse solenocytes were used as responder cells in the mitogen proliferative assay. The proliferative responses were assessed by CFSE labeling and FACS. (Inside the gray line, there are unstimulated splenic cells.) (*n* = 3, *P < 0.05 versus mDC; #*P* < 0.05 versus NC-HGF-MSC; &*P* < 0.05 versus NC-shHGF-MSC; data are expressed as mean ± SD). Each experiment was repeated at least three times

To investigate whether the differentiation of mDCs into DCregs induced by MSCs is affected by HGF secretion, we again performed a DC phenotypic analysis to show that HGF-MSC-treated mDCs expressed fewer functional markers such as MHCII and CD86 than MSC-DCs, but more than shHGF-MSC-treated mDCs (Fig. 4a). DCs were then independently cultured for another 24 h after thorough washes, and we observed that IL-10 and TGF-β were increased in the MSC-DC and HGF-MSC-DC groups, and IL-12 was decreased. After knocking down the HGF gene in MSCs, the regulatory effect of MSC-DCs on the above cytokines was weakened (Fig. 4b). In addition, compared with mDCs, the DCs cocultured with MSCs had stronger phagocytic capacity, and HGF-MSC-DCs had the strongest, followed by MSC-DCs and then shHGF-MSC-DCs (Fig. 4c). To investigate whether the ability of HGF-MSCs/shHGF-MSC-DCs to stimulate lymphocyte proliferation was different from that of mDCs and MSC-DCs, a mitogen proliferative assay was performed. CFSE-labeled splenic lymphocytes were used as responders and cocultured with allogenic mDCs, MSC-DCs HGF-MSC-DCs, and shHGF-MSC-DCs. There was a gradient in lymphocyte proliferation according to the type of MSCs, with the greatest response observed in coculture with mDCs, then shHGF-MSC-DCs and MSCs, and the least difference between groups for HGF-MSC-DCs (Fig. 4d, e). These results demonstrate that the HGF secreted by MSCs induces mDCs into immune-tolerant DCs, inhibits lymphocyte proliferation, and regulates the release of inflammatory cytokines.

### HGF induces the differentiation of mDCs into DCregs via the AKT signaling pathway

**Fig. 5:**
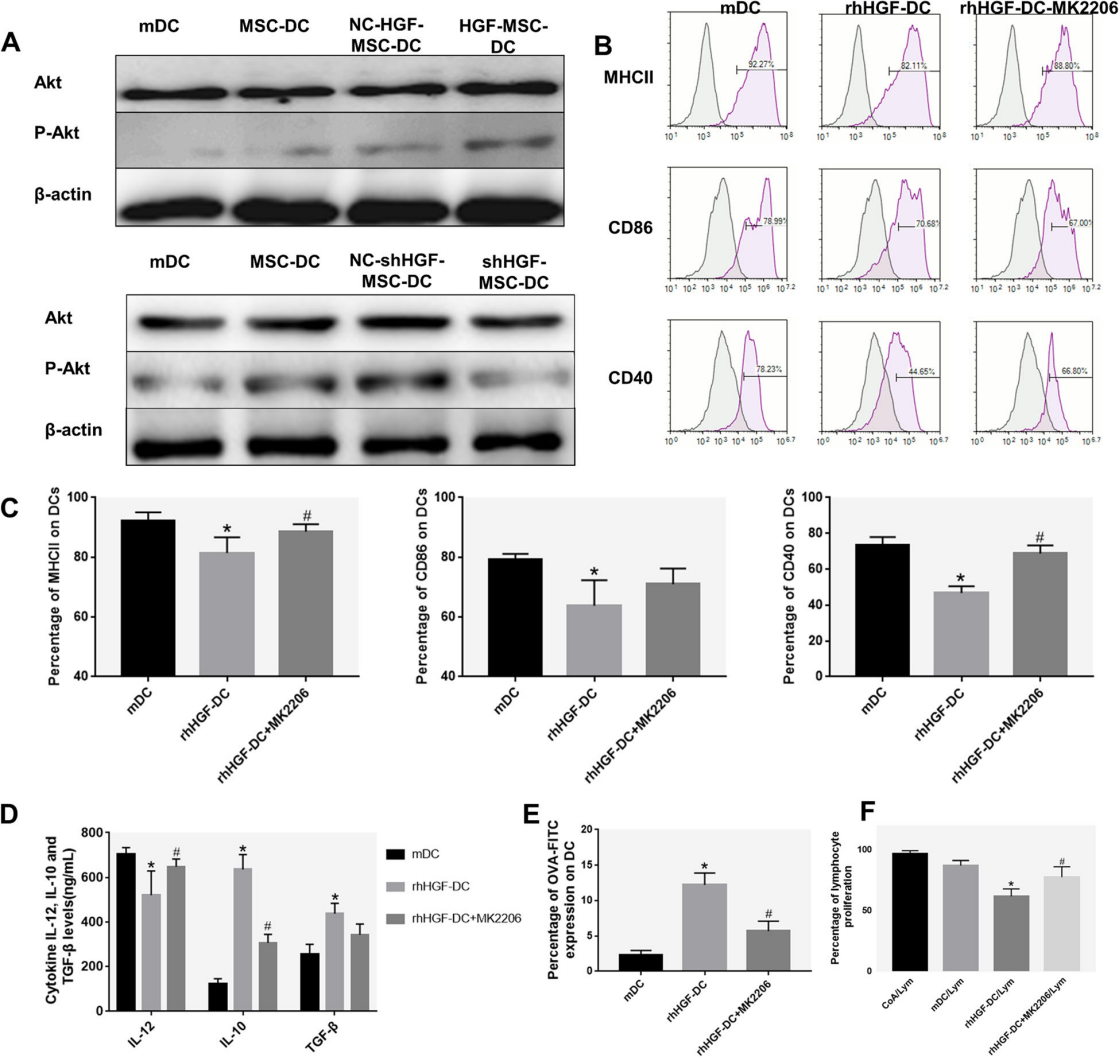
The role of the AKT pathway in MSC-secreted HGF induction of DC differentiation. **a** The expression of Akt protein and Akt phosphorylation levels in DCs cultured for 72 h in the presence of MSCs or MSCs after transduction was evaluated using Western blot analysis. **b**, **c** Immunophenotype analysis of DCs (expression of MHCII, CD86, and CD40 in mDCs cultured for 72 h in the presence of rhHGF or MK2206 and rhHGF). **d** Cytokine secretion profiles of DCs cultured for 72 h in the presence of rhHGF or MK2206 and rhHGF. **e** Phagocytic ability of HGF-induced DCs (percentage of OVA-FITC-positive cells). **f** Lymphocyte proliferation stimulated by ConA, mDCs, rhHGF-DCs, and rhHGF + MK2206-DCs. Normal BALB/c mouse solenocytes were used as responder cells in the mitogen proliferative assay. The proliferative responses were assessed by CFSE labeling and FACS (*n* = 3, *P < 0.05 versus mDC; #*P* < 0.05 versus rhHGF-DC; data are expressed as mean ± SD). Each experiment was repeated three times

To directly examine the mechanism of Akt in DCreg generation, we tested the phosphorylation level of the Akt protein by Western blot. Surprisingly, we observed that mDCs cocultured with MSCs had more p-Akt molecules than mDCs. When mDCs were cocultured with HGF-MSCs, Akt phosphorylation increased, while it decreased when mDCs were cocultured with shHGF-MSC (Fig. 5a). Collectively, these data show that the HGF effects are associated with the stimulation of Akt signaling during the differentiation of mDCs into DCregs.

Furthermore, we added AKT inhibitor (MK-2206) in the rhHGF and DC culture system and observed that the immunomodulatory effects of rhHGF were significantly attenuated in the Akt inhibitor group. Higher levels of MHCII, CD86, and CD40 were expressed in the Akt inhibitor group compared with the rhHGF-DC group (Fig. 5b, c). HGF reduced the secretion of IL-12 and increased the secretion of IL-10 and TGF-β in DCs, and these immunomodulatory effects were inhibited by MK-2206 (Fig. 5d). Inhibition of Akt increased the DC phagocytosis and lymphocyte proliferation stimulated by treatment with HGF (Fig. 5e, f). These results indicate that MK-2206 weakens the regulatory effect of HGF and that Akt activation is involved in the immune regulation of DCs.

### The tolerogenic DCs induced by MSC-secreted HGF attenuate ALI

**Fig. 6:**
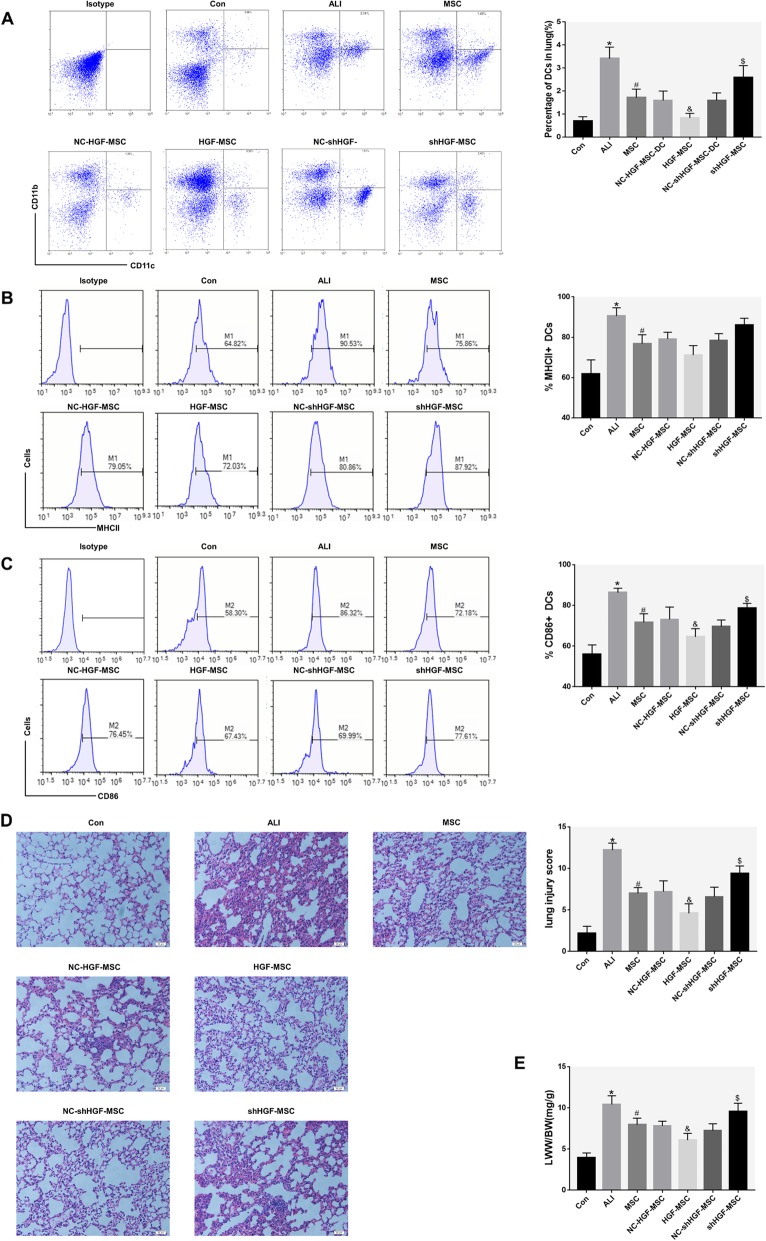
Effects of MSCs on pulmonary DCs in ALI mice by secreting HGF. **a** Immunophenotype analysis of pulmonary DCs in ALI mice treated with transfected or untransfected MSCs. Representative dot plots and bar graph showing the percentage of respiratory cDCs (gate on the upper right quadrant) in the presence of normal saline, MSCs, NC-HGF-MSCs, HGF-MSCs, NC-shHGF-MSCs, and shHGF-MSCs. **b** Charts and bar graph showing the expression of MHCII on respiratory cDCs (gate on the right in each panel, M1). Pulmonary cDC phenotype changes were statistically analyzed. **c** Charts and bar graph showing the expression of CD86 on respiratory cDCs (gate on the right in each panel, M2). Pulmonary DC phenotype changes were statistically analyzed. **d** Representative histology sections of lung tissue at 24 h (hematoxylin and eosin staining; magnification, ×200) and the pathological lung injury score at 24 h. **e** Comparison of the lung wet weight to body weight ratio (LWW/BW) in different groups at 24 h. These data are expressed as the mean ± SD (*n* = 5, for each group at 24 h, *P < 0.05 versus the Con group; #*P* < 0.05 versus the ALI mice, &*P* < 0.05 versus the NC-HGF-MSC mice; $*P* < 0.05 versus the NC-shHGF-MSC mice; data are expressed as the mean ± SD)

To verify the regulation of MSCs on lung DCs in ALI mice and determine if its mechanism is related to the secretion of HGF, we treated lung DCs with MSCs, HGF-MSCs, and shHGF-MSCs 6 h after ALI model establishment and compared their effects on mDCs. In unchallenged mice, the frequency of CD11c^+^ CD11b^+^ cDCs in the lung tissue was low, while the frequency of lung cDCs was significantly increased 24 h after airway injection of LPS and was reduced after treatment with MSC. Treatment of ALI mice with HGF-MSCs further reduced DC aggregation, while treatment with shHGF-MSCs increased lung DC aggregation (Fig. 6a). These results demonstrate a rapid increase in total lung cDC recruitment in LPS-induced ALI mice and show that MSCs inhibit lung DC aggregation by secreting HGF.

MDCs are characterized by a further increase in the expression of the stimulatory molecules CD40, CD80, CD86, and MHCII on their surface. We analyzed the maturation status of lung cDCs based on the expression of CD86 and MHCII. At baseline, lung cDCs in the control group at 24 h expressed relatively low levels of CD86 and MHCII (Fig. 6b, c). In parallel to the accumulation of pulmonary cDCs in ALI mice, the expression of CD86 and MHCII on the surface of respiratory cDCs showed a significant increase. Notably, treatment with MSCs led to a marked reduction in CD86 or MHCII expression on pulmonary cDCs. Treatment with HGF-MSCs resulted in a significant reduction in CD86 on lung cDC compared to treatment with the empty vector control group but increased after treatment with shHGF-MSCs (Fig. 6c). Then, we used a histological evaluation of the lungs to confirm the effect of different MSC treatments on LPS-induced lung injury in mice (Fig. 6d). Lung specimens from ALI mice showed extensive 24-h alveolar wall thickness caused by edema, as well as significant inflammatory cell infiltration that were improved after MSC treatment. High expression of the HGF gene promoted MSCs to alleviate lung pathological damage, and knockdown of the HGF gene expression inhibited the effect of the MSC treatment (Fig. 6d). The LWW/BW response to pulmonary edema is related to the severity of lung injury [35]. The LWW/BW in ALI mice was significantly higher than that in control mice at 24 h, indicating that LPS administration effectively induced ALI (Fig. 6e). In contrast, when ALI mice were treated with MSC, the LWW/BW was significantly lower than that of ALI. Treatment with HGF-MSC resulted in a significant reduction in LWW/BW compared to the empty vector control group but increased after treatment with shHGF-MSCs (Fig. 6e). These results indicated that MSCs are dependent on HGF to attenuate LPS-induced ALI.

## Discussion

Here, we uncover that MSCs alleviate ALI via paracrine HGF regulation of DC immune function. Our findings suggest that imDCs are induced to mature after 24 h of LPS (50 ng/ml) stimulation. Via the HGF/Akt pathway, MSCs induced mDCs to differentiate into DCregs that have low expression of MHCII, CD86, and CD40 molecules, strong phagocytic function, and weakened promotion of T cells. MSCs decreased lung DC aggregation and maturity and alleviated lung pathological injury in LPS-induced ALI mice. High expression of the HGF gene enhanced the above effects of MSCs, while decreased expression of HGF weakened the above effects of MSCs.

The induction of mDCs into DCregs is more likely to be the key to early ALI treatment. DCs are present throughout the lung tissue, underlying the epithelial layer, and under steady-state conditions have fewer numbers and immature phenotypes and are ready to encounter foreign material, infection, or tissue damage [16, 36]. After inhalation of bacterial stimulation, airway mucosal DCs accelerate antigen sampling and transport, reaching regional lymph nodes and expressing complete APC activity within 30 min after microbial exposure [37]. DCs rapidly accumulate in the lungs, reaching a peak within 2 h and maturation within 6 h after the inhalation of pathogenic substances [14, 38]. Our study found that the accumulation of DCs in mouse lungs remained significant 24 h after inducing ALI, and the in vitro experiments also showed that the phagocytosis of DCs after LPS stimulation was significantly reduced. Once mobilized into the lungs, the DCs sample the incoming antigen and undergo a maturation process characterized by the upregulation of cell surface expression of MHCII and costimulatory molecules (e.g., CD80 and CD86) [14]. Similar phenotypes were detected in the lungs of ALI mice in this study. ARDS is an ALI mediated by an uncontrolled inflammatory response [39], and mDCs play an important immunomodulatory role during the ARDS exacerbation process [16, 36]. Therefore, regulating the differentiation of mDCs into DCregs may improve ALI.

Studies have shown that mDCs are induced by MSCs to differentiate into DCregs with reduced expression of MHCII, CD11c, CD80, CD86, and CD40 [18, 19], but whether this phenomenon regulates the immune response in ARDS is unclear. The results showed that MSCs may induce mDCs into a novel DCreg population with high endocytosis and downregulated expression of the costimulatory molecules MHCII, CD86, and CD40. LPS stimulation did not reverse this trend, demonstrating that a different DC population was induced by MSCs. This is consistent with previous reports [19]. Our further in vivo study showed that the number of conventional DCs and mDCs in the lung decreased and lung injury and pulmonary water content were reduced in the MSC-treated group compared with the ALI group. Our data demonstrated that MSCs induces the transformation of mDCs into DCregs to reduce ALI. However, the mechanism by which MSCs regulate DCs to treat early ALI remains unclear.

Paracrine HGF is an important mechanism by which MSCs regulates DCs to alleviate ARDS lung injury. Since the discovery and characterization of the epithelial specific growth factors keratinocyte growth factor (KGF) and HGF, their role in lung development, lung inflammation, and repair has been extensively studied [40, 41]. Our previous research also showed that the ability to express HGF was required for MSCs to protect the injured lung [26]. However, an RCT by McAuley DF et al. found that KGF was not beneficial for physiological outcomes in ARDS and could make clinical outcomes worse [41], which was quite different from the results of animal experiments [42 43]. The reasons may be as follows: the effect of giving KGF before lung injury is better than that after injury, local administration of intratracheal is better than systemic [42], heterogeneity for different reasons makes ARDS patients to have different effects on treatment, or exogenous KGF supplementation is not related to any other beneficial effects. In addition, unlike KGF, others and our studies have shown that in addition to repairing epithelial damage, HGF not only has the effect of repairing epithelial damage but also repairs endothelial damage and immune regulation [26, 29, 43, 45]. Consistent with previous reports, HGF increased the expression of PD-L1 on DCs that mediate regulatory T cell induction and tolerance [28, 44]. Our results also indicate that MSC or HGF treatment significantly increased PD-L1 expression in DCs. Therefore, HGF is more likely to be an ARDS treatment option than KGF. HGF produced by MSCs also mediates T cell suppression [45] and is involved in the inhibition of imDC activation [46]. Studying the process of MSC-induced DC immune tolerance, we also unexpectedly found that the phosphorylation level of the HGF receptor c-Met protein in DCs was significantly increased, so we speculated that HGF/c-met may be an important link in the MSC-mediated DC production. Further verification results showed that HGF could induce mDCs to produce an immunotolerant phenotype, such as MSC-DC, and HGF-DCs can also reduce lymphocyte proliferation and IL-12 secretion and increase TGF-β and IL-10 production compared with mDCs. These results revealed that MSC-induced DC immune tolerance may be related to HGF secretion.

That MSCs alleviate lung injury by secreting HGF has been confirmed in vivo and in vitro [26, 47], but whether it is related to DCreg generation is still unknown. Compared with NC-HGF-MSC-DCs, DCs obtained by coculture with the HGF overexpressing HGF-MSCs had lower levels of mDC markers, IL-12 secretion, and ability to induce the proliferation of lymphocytes after stimulation, but TGF-β and IL-10 increased more significantly. In contrast, the immunosuppressive effects of MSCs on mDCs were downregulated in the shHGF-MSC group. Thus, shHGF-MSC-DCs expressed higher levels of mature functional markers, secreted higher levels of IL-12, and induced the proliferation of more lymphocytes after coculture and had a larger decrease in TGF-β and IL-10. The secretion of inflammatory cytokines was consistent with previous studies. MSCs inhibited the secretion of the pro-inflammatory factor IL-12 [48], promoting the secretion of the anti-inflammatory factor TGF-β and IL-10 [26, 49]. As expected, in animal studies, compared with the MSC treatment group, treatment MSCs with an upregulated HGF secretion led to improvement of acute lung inflammation and lung injury and promoted mDCs into DCregs in the murine model of LPS-induced ALI, but the opposite results were observed in the shHGF-MSC treatment group. The results indicated that MSCs were partially dependent on the secretion of HGF to induce mDC immune tolerance for the treatment of early ALI, but the downstream mechanism of HGF was still unclear.

Previous studies have shown that HGF can inhibit the activation of conventional DCs via the c-Src-PI3K-AKT-mTOR-GSK3β pathway [46, 50]. Singhal et al. found that proximal signal transduction events induced in dendritic cells by HGF include the physical association of c-Src with the HGF receptor c-MET and the associated activation of c-Src. Activation of c-Src, in turn, establishes a complex of phosphatidylinositol 3-kinase and c-MET and promotes downstream activation of phosphatidylinositol 3-kinase/AKT (Ser473). Notably, activation of c-Src stimulated by HGF leads to induction of phosphatidylinositol 3-kinase complexes p85α/p110α and p85α/p110δ, which are necessary to activate the target of rapamycin [46]. This study found similar results that the phosphorylation levels of c-Met and Akt (Ser473) were significantly increased in MSC-treated DCs. Therefore, this result suggests that the c-Met/Akt pathway is involved in the HGF mechanism of inducing mDC immune tolerance production. The phosphorylation of c-Met and Akt in DCs was positively correlated with HGF expression in MSCs, indicating that c-Met/Akt is located downstream of HGF during the induction of DCreg production. In this study, the expression of costimulatory molecules in DCs and functional markers of T cells stimulated by DCs showed that HGF could induce DCreg production, and Akt inhibitors could reverse the result. Consequently, these results revealed that the disruption of Akt phosphorylation at Ser473 downregulated the effect of HGF on DC regulation. Thus, these data suggest that MSCs might activate the HGF/Akt pathway, promoting the differentiation of mDCs into DCregs to reduce ALI, but the mechanism downstream of AKT remains to be elucidated in future experiments. There has been a lot of progress in the knowledge of the upstream regulatory inputs into AKT, but key multi-function downstream signaling nodes (GSK3, FoxO, mTORC1) greatly expand the functional range of AKT [51]. According to previous studies [46], HGF treatment caused sequential activation cascade c-Src → PI3K → AKT → mTOR in DCs, and activation of mTOR through the pathway c-Src-PI3K-AKT depends on HGF-stimulated GSK3 beta inactivation. This may be an important mechanism by which MSC secretes HGF-induced DC to alleviate ALI.

## Conclusions

This study showed that the induction of mDC immune tolerance is an important process for MSCs to alleviate early ALI, and this process is closely related to paracrine HGF. These findings provide new insights into the role of MSCs in the secretion of HGF to repair lung injury. We believe that these studies will enhance our understanding of ARDS/ALI immunotherapy.

## Data Availability

All datasets used and/or analyzed during the current study are available from the corresponding author on reasonable request.

## Abbreviations

Abs: Antibodies
ALI: Acute lung injury
ANOVA: Analysis of variance
ARDS: Acute respiratory distress syndrome
BM: Bone marrow
BSA: Bovine serum albumin
cDCs: Conventional DCs
CFSE: Carboxyfluorescein diacetate succinimidyl ester
ConA: Concanavalin A
DCregs: Regulatory DCs
DCs: Dendritic cells
ECL: Enhanced chemiluminescence
EGFP: Enhanced green fluorescent protein
ELISA: Enzyme-linked immunosorbent assay
FBS: Fetal bovine serum
GM-CSF: Granulocyte-macrophage colony-stimulating factor
HGF: Hepatocyte growth factor
IL-10: Interleukin-10
IL-12: Interleukin-12
IL-4: Interleukin-4
imDCs: Immature DCs
LPS: Lipopolysaccharide
LWW/BW: Lung wet weight to body weight ratio
mDCs: Mature DCs
MSC: Mesenchymal stem cell
PBS: Phosphate-buffered saline
PD-L1: Programmed death ligand 1
PVDF: Polyvinylidene difluoride
SD: Standard deviation
TGF-β: Transforming growth factor beta
